# Impact of bounded noise on the formation and instability of spiral wave in a 2D Lattice of neurons

**DOI:** 10.1038/srep43151

**Published:** 2017-02-21

**Authors:** Yuangen Yao, Haiyou Deng, Ming Yi, Jun Ma

**Affiliations:** 1 Department of Physics, College of Science, Huazhong Agricultural University, Wuhan 430070, China; 2 Institute of Applied Physics, Huazhong Agricultural University, Wuhan 430070, China; 3 Department of Physics, Lanzhou University of Technology, Lanzhou 730050, China; 4 NAAM-Research Group, Department of Mathematics, Faculty of Science, King Abdulaziz University, P.O.Box 80203, Jeddah 21589, Saudi Arabia

## Abstract

Spiral waves in the neocortex may provide a spatial framework to organize cortical oscillations, thus help signal communication. However, noise influences spiral wave. Many previous theoretical studies about noise mainly focus on unbounded Gaussian noise, which contradicts that a real physical quantity is always bounded. Furthermore, non-Gaussian noise is also important for dynamical behaviors of excitable media. Nevertheless, there are no results concerning the effect of bounded noise on spiral wave till now. Based on Hodgkin-Huxley neuron model subjected to bounded noise with the form of *A*sin[*ωt* + *σW(t*)], the influences of bounded noise on the formation and instability of spiral wave in a two-dimensional (2D) square lattice of neurons are investigated in detail by separately adjusting the intensity *σ*, amplitude *A*, and frequency *f* of bounded noise. It is found that the increased intensity *σ* can facilitate the formation of spiral wave while the increased amplitude *A* tends to destroy spiral wave. Furthermore, frequency of bounded noise has the effect of facilitation or inhibition on pattern synchronization. Interestingly, for the appropriate intensity, amplitude and frequency can separately induce resonance-like phenomenon.

The neuronal system, which consists of a huge number of neurons, plays a pivotal role in regulating physiological behaviors^1^,^2^. Neurons in the neuronal system are often coupled to form nonlinear complex networks. Due to the importance in theoretical studies and potential medical applications, the collective behaviors of neurons in networks, such as formation and instability of patterns, have been studied extensively^3^,^4^,^5^. Various spatiotemporal patterns are developed in the neuronal networks during the signal propagation in the media, and the collective electrical behaviors of neurons in networks often manifest spatiotemporal patterns^6^,^7^. Spiral wave, one of spatiotemporal patterns, had been observed experimentally in the mammalian neocortex^4^,^8^,^9^,^10^. The spiral waves in the neocortex provide a spatial framework to organize cortical oscillations, thus play important role in signal communication by coordinating oscillation phases over a group of neurons^4^ like pacemaker. On the other hand, spiral wave in the neocortex may contribute to seizure generation in pathological conditions by extending the duration of evoked activity and interacting with incoming input signals^4^. Except in the neuronal system, spiral wave is also observed in the cardiac tissue, which is harmful and related to a kind of heart disease, such as heart ventricular fibrillation^11^. Breakup of spiral can result in rapid death of heart on account of ventricular fibrillation^11^. Therefore, the suppression of spiral wave in the cardiac tissue is beneficial to preventing ventricular fibrillation^11^. Furthermore, spiral waves also exist in other biological systems, such as retinal spreading depression^12^, fertilizing Xenopus oocyte^13^, etc. The formation, instability and dynamics of spiral waves in biological systems are important in its biological function. Instability and breakup of spiral wave in the biological networks can make disorder and possible collapse. As a consequence, it is particularly intriguing to investigate the formation, instability and dynamics of spiral waves in neuronal networks with different topologies and driven by various signals and noises. In fact, spiral wave is a basic characteristics of excitable systems, which have been observed widely in a wide variety of nonlinear physical, chemical^14^ and biological systems^4^. Hence, the related studies about spiral waves also facilitate a better understanding of nonlinear dynamics.

Noise is inevitable in neuronal systems, and is intuitively harmful due to destroying synchronization in coupled neuron network^15^. However, many research results in theoretical analysis, numerical simulations and experimental work have revealed the counterintuitive influences of noise. That is, noise with appropriate intensity can play a constructive role in large variety of nonlinear systems^16^,^17^. Stochastic resonance (SR) and coherence resonance (CR) are two major noise-induced phenomena. SR is noise-induced enhancement of regularity in nonlinear systems, which appears when noise and a periodic signal simultaneously drive a nonlinear system^18^,^19^. In other words, noise can amplify and optimize generally weak periodic input signal, and the response of the system shows resonance-like behavior as the change of noise amplitude^19^. Contrary to SR, even in the absence of external periodic driving, characteristic correlation time of the noise-excited oscillations in the nonlinear system can reach to maximum at certain noise level, and coherent oscillations are observed. Therefore, the coherence appears as a nonlinear response to purely noisy excitation. This phenomenon is known as CR^18^. The ubiquitous and inevitable noise has a very important impact on formation, instability and dynamics of pattern in the neural network^5^,^20^,^21^. There are many examples demonstrating that appropriate noise intensity can enhance pattern formation of neuron network by the mechanism of CR or SR. For instance, Gaussian white noise can induce SR in the Hindmarsh–Rose system^22^ and CR in the FitzHugh–Nagumo system^18^. Besides noise-induced patterns, noise-induced synchronizations are also widely observed in neuronal network^23^. The influences of various different noises, such as Gaussian white^24^ and colored noise^25^, channel noise^26^, on spiral wave are also widely explored in our previous studies. Our results further confirm that too weak or too strong noise may act against the formation of spiral waves, and appropriate noise can facilitate formation and development of spiral wave^25^. Meanwhile, we also found that spiral wave is robust below a certain threshold of noise intensity; otherwise, the breakup of spiral wave occurs over this threshold^27^. Therefore, it is an interesting topic to search the optimized noise intensity.

The formation and stability of spiral wave is the base for stably signal communication by virtue of spiral waves. However, noise-induced instability of spiral waves may interrupt signal propagation^27^. It is important to generate stable rotating spiral wave and investigate the impact of noise on the spatiotemporal dynamics of spiral waves. Although a number of efforts have been contributed to this area, Gaussian noises are adopted in many studies for convenience of analysis. Gaussian noise has a constant power spectral density in a range of infinity bandwidth^28^, and has the probability of taking large values^29^. In other words, Gaussian noise is unbounded, which violates that a real physical quantity is always bounded^29^. Gaussian noise is inappropriate to investigate the random effects in some real physical or biological systems^30^,^31^,^32^. For example, the use of Gaussian noise in anti-tumor chemotherapy may result in biologically paradoxical results, and suitable bounded noise is adopted instead of Gaussian noise^31^. When investigating a neuronal network under random disturbance, it is important to choose appropriate noise, whose statistical and probabilistic properties are in accord with real random process^29^. Many experimental studies in sensory and other biological systems have shown that it is necessary to investigate effect of non-Gaussian noises^33^. As a typical kind of non-Gaussian noise, bounded noise is very simple in mathematical presentation, which only consists of harmonic function with constant amplitude and random frequency and phase^34^. But bounded noise is a suitable model for common random fluctuating^35^. It has been used for a long time in electrical engineering, recently used in mechanical and structural engineering systems, even in biological systems^30^,^33^,^34^,^36^. At the biochemical and biophysical level there are many noise sources in neurons, such as the opening and closing of ion channels, the diffusion and binding of signal molecules to receptors^37^. Moreover, neurons communicate with each other by transmitting various signals. The phase of signals may vary randomly with time, which may result in this kind of noise. For example, phase shift may occur when a periodic wave travels through a random medium. Recently, many interesting studies associated with bounded noise have been carried out in neuronal systems^28^,^38^,^39^. Similar to Gaussian noise, CR is also observed in the neuron network driven by bounded noise^28^. Moreover, Yang and co-workers investigated the impact of bounded noise and shortcut on the spatiotemporal dynamics of small-world neuron network of two-variable Terman–Wang neuron model^39^, and they founded that noise always impair spatial synchronizability among coupled neurons, and yet CR occurs at an appropriately noise level^39^. However, as we known, there has been no attention and related studies to the effect of bounded noise on the formation, instability and dynamics of spiral wave. Therefore, it is very necessary to investigate spiral waves in neuronal network subjected to bounded noises.

Motivated by the previous findings and the importance of spiral wave, bounded noises with sine-function form are imposed on some neurons, whose local kinetics is described by the famous Hodgkin-Huxley (H-H) neuron model, while neurons are coupled with nearest-neighbor connection to form a two-dimensional (2D) square lattice. Then the effect of bounded noise on the formation, instability and dynamics of spiral wave are investigated in detail in this study from non-linear dynamic point of view.

## Bounded noise and neural model for 2D lattice network

Before introducing the model for 2D lattice neuronal networks, bounded noise is firstly represented as follow^28^,^34^,^39^:


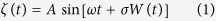


Herein, *A* and *ω* denote the amplitude and angular frequency of bounded noise, respectively. *W (t*) is a unit Wiener process, while *σ* represents the intensity of the unit Wiener process *W (t*). For *t* → ∞, the mean, autocorrelation function, and power spectral density of bounded noise are^34^,^39^


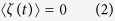



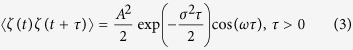



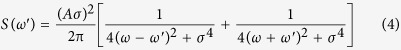


According to equation (4), it is obvious that power spectrum of bounded noise depends on the parameters of *A, ω* and *σ* synchronously. And it shows two symmetrical peaks at *ω*′ = ±*ω*. When *ω* → 0, the two peaks merge into one, and the bounded noise *ζ (t*) becomes the sine-Wiener noise. Moreover, the bandwidth of *ζ (t*) is dominated by the parameter *σ*. Bounded noise can approach white noise when *σ* → ∞, while it turns into narrow-band process when *σ* is small enough. Furthermore, the bounded noise *ζ (t*) is a sinusoidal periodic signal when *σ* = 0. As previously mentioned in ref. 40, the increment of standard Wiener process is generated in our numerical simulation by the following formula:


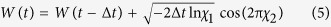


where Δ*t* indicates time step, and *χ*_1_, *χ*_2_ are two independent random numbers between 0 and 1 with equal probability.

The dynamical model for 2D lattice network of H-H neurons^41^,^42^ is given by:


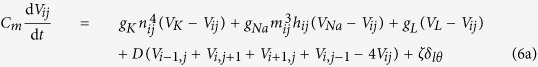



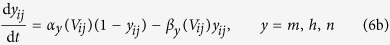


Here, the *δ* function defines the range of grids driven by bounded noise, which let the bounded noise being local. For simplicity, the bounded noises are only imposed on the neurons located on the left hemi lattice.


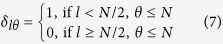


And the channel parameters of *α*_*y*_ and *β*_*y*_ are decided by ref. 42:


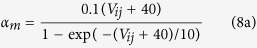



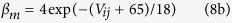



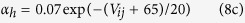



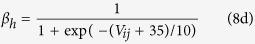



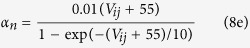



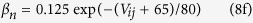


Herein, the subscripts *i* and *j* of the variables describes the position of the neurons in the network. *V*_*ij*_ is the voltage of the cellular membrane of the neuron at the node (*i, j*), while *m*_*ij*_, *n*_*ij*_ and *h*_*ij*_ are parameters for gate channel of the neuron at the node (*i, j*). The capacitance of membrane is *C*_*m*_ = 1 μF/cm^2^. The maximal conductance of potassium and sodium are *g*_*K*_ = 36 mS/cm^2^ and *g*_*Na*_ = 120 mS/cm^2^, respectively. The conductance of leakage current is *g*_*L*_ = 0.3 mS/cm^2^. The reversal potential of potassium, sodium and leakage current are *V*_*K*_ = −77 mV, *V*_*Na*_ = 50 mV and *V*_*L*_ = −54.4 mV, respectively^42^. Unless otherwise specified, the coupling coefficient *D* between neurons is 0.5. Time step Δ*t* = 0.001 ms, neurons number *N* × *N* = 100 × 100, the Euler forward difference procedure and no-flux boundary condition are used in our numerical simulation^27^. Spiral waves can be induced and developed by many schemes. Here specific initial values can be used to trigger a spiral seed in small central field of lattice^27^, and then the perfect spiral wave can be induced and developed in entire network with the appropriate excitability^27^.

To quantitatively study the collective behaviors and statistical properties of spiral wave during evolution processes, a factor of synchronization *R* is defined to characterize spatial synchronization of the collective spikes^43^, based on the mean-field theory.


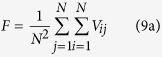



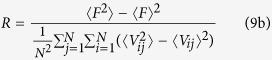


where, *N*^2^ is the total number of all neurons. *V*_*ij*_ is the membrane potential of the neuron at the node (*i, j*). Moreover, the symbol of 〈*〉 represents averaging over time. Clearly, larger *R* indicates better synchronization. *R* is very close to 1 indicates perfect synchronization, while *R* approaches zero for no synchronization. As our previous studies, synchronization factor *R* can be used to detect the critical bifurcation parameters which cause the striking change of pattern, such as breakup or elimination of the spiral wave^43^. Namely, the sudden change of curve of *R* vs. bifurcation parameter manifests the phase transition.

## Main results

### Influences of intensity on formation and instability of spiral wave

**Figure 1:**
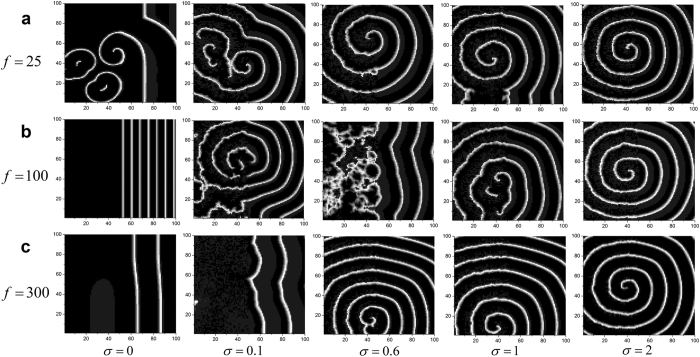
Developed patterns in the network at *t* = 500 time units, which are obtained by adjusting intensity *σ* for a fixed amplitude *A* = 20 and the different frequencies of (**a**) *f* = 25; (**b**) *f* = 100 and (**c**) *f* = 300.

Firstly, we discuss how the intensity *σ* of the unit Wiener process changes the wave formation and instability in the network at the different frequencies of bounded noise. Some developed spiral waves are illustrated in Fig. 1 for the different intensities *σ* and frequencies *f* when we set *A* = 20. For *f* = 25, multi-spiral waves with incomplete wave-shape maybe coexists when *σ* is set to the lower values, such as 0 or 0.1. However, as *σ* is gradually increased, only one spiral wave with complete wave-shape can be observed (Fig. 1a). This indicates that there may be a transition between single- and multi-spiral waves for the appropriate parameter values. For *f* = 100, only plane wave exists at the half right of lattice when *σ* = 0. As *σ* is increased, such as 0.1, the shape of spiral wave is being gradually formed. However, the spiral wave is under instability, and it maybe breaks again subjected to the perturbation of noise. Finally, the steady and stable spiral wave can be induced when *σ* is large enough, such as 2 (Fig. 1b). For the case of *f* = 300, with the gradually increase of *σ*, the shape of spiral wave gradually produces, the steady and stable spiral wave can be finally induced (Fig. 1c). Taken together, these figures indicate that noise can facilitate the development of spiral wave in the network.

**Figure 2:**
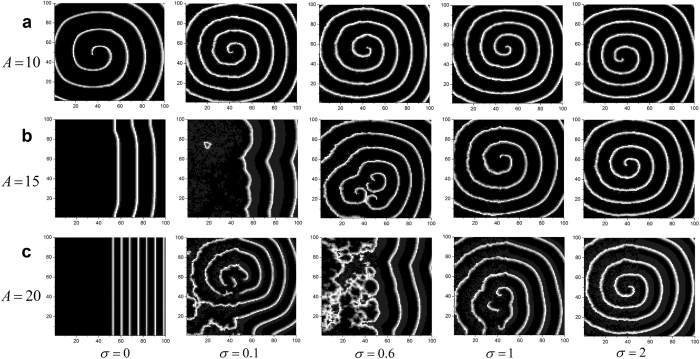
Developed patterns in the network at *t* = 500 time units, which are obtained by adjusting intensity *σ* for a fixed frequency *f* = 100 and the different amplitudes of (**a**) *A* = 10; (**b**) *A* = 15 and (**c**) *A* = 20.

Next, we investigate how the intensity *σ* of the unit Wiener process affects wave formation and instability in the network at the different amplitudes of bounded noise. To this purpose, we also illustrate some developed spiral waves in Fig. 2 for the different intensities *σ* and amplitudes *A* when we set *f* = 100. For the lower amplitude, e.g. *A* = 10, as *σ* is increased, spiral wave always exists, and gradually becomes dense (Fig. 2a). With the increase of *A*, spiral wave maybe be interrupted or does not generate (Fig. 2). Thus, increased amplitude *A* tends to destroy spiral wave. However, the augmenting *σ* can induce spiral wave again (Fig. 2c).

**Figure 3:**
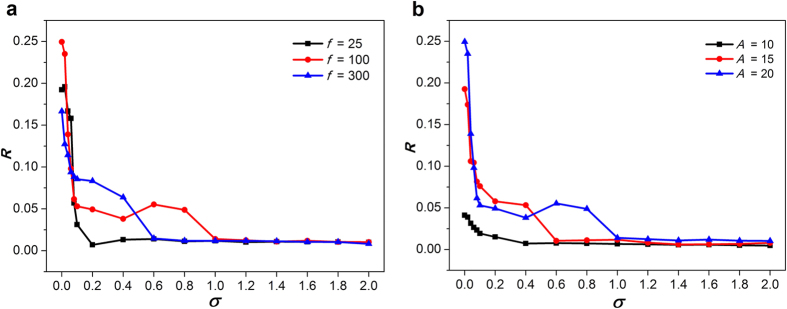
Synchronization factor *R* as a function of intensity *σ* (**a**) for a fixed amplitude *A* = 20 and the different frequencies; (**b**) for a fixed frequency *f* = 100 and the different amplitudes. We set sufficiently large 500 time units in the calculation of *R*.

To further confirm the above observed phenomena quantitatively, the factor of *R* vs. increasing *σ* is plotted for the different frequencies (Fig. 3a) or for the different amplitudes (Fig. 3b). According to the previous studies, the curve of *R* may not be able to finely describe the formation and dynamics of patterns, but the sudden change of curves indeed indicates the phase transition of patterns, and the smaller *R* indicates high probability to induce spiral wave^43^. As a whole, *R* decreases gradually along with the increase of *σ*, which validates that spiral wave can be induced by the strengthening of *σ* (Fig. 3). In other words, the spatial synchronization in this 2D lattice neuronal networks is generally degraded upon the increasing *σ*. This is in accordance with the results shown in Figs 1 and 2. Moreover, for *f* = 100, a little peak appears between 0.4 and 1 of *σ* (Fig. 3a,b), which indicates resonance-like phenomenon (Fig. 1b).

### Influences of amplitude on formation and instability of spiral wave

**Figure 4:**
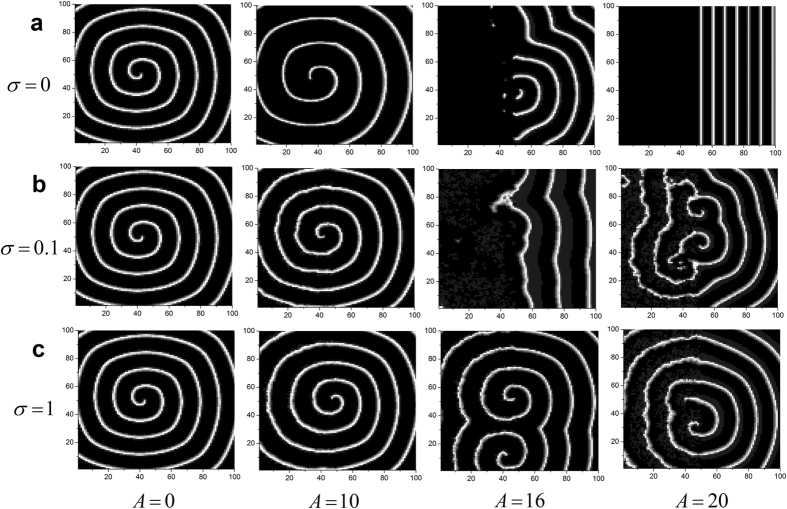
Developed patterns in the network at *t* = 500 time units, which are obtained by adjusting amplitude *A* for a fixed frequency *f* = 100 and the different intensities of (**a**) *σ* = 0; (**b**) *σ* = 0.1 and (**c**) *σ* = 1.

It is also interesting to check influences of amplitude on formation and instability of spiral wave. In what follows, we investigate how amplitude influences wave formation and instability in the network. To begin with, some developed spiral waves are illustrated in Fig. 4 when we set *f* = 100. For *σ* = 0, the spiral wave become sparse, and then eventually break due to enhancement of amplitude (Fig. 4a). When *σ* and *A* are set to 0 and 16, respectively, it becomes very obvious that bounded noise erases the left hemi pattern of spiral wave (Fig. 4a). The similar conclusion can be also observed for the larger *σ*, such as 0.1 or 1 (Fig. 4b,c). For the case of *σ* = 0.1, the phase transition of pattern occurs in the parameter region from *A* = 10 to 20. Moreover, there is also a transition from multi-spiral waves to single-spiral wave at appropriate parameters for *σ* = 1. Judging from transverse direction of Fig. 4, larger amplitude *A* generally tends to destroy the pattern of spiral wave, while intensity *σ* can induced or stabilize spiral wave from the vertical point of view (Fig. 4). Furthermore, the various complex dynamics, such as phase transitions from stable to instability, instability to stable, and multi-to-single spiral wave, can occur by controlling both parameters of *A* and *σ* (Fig. 4).

**Figure 5:**
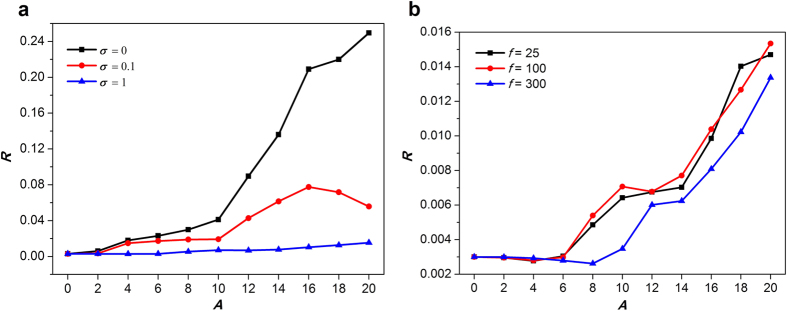
Synchronization factor *R* as a function of amplitude *A* (**a**) for a fixed frequency *f* = 100 and the different intensities; (**b**) for a fixed intensity *σ* = 1 and the different frequencies. We set sufficiently large 500 time units in the calculation of *R*.

The curve of *R* vs. increasing *A* is drawn to give a quantitative understanding of the above findings (Fig. 5). For *σ* = 0, amplitude is always helpful for synchronization of whole network because larger *R* indicates better synchronization. For *σ* = 0.1, the dependence of *R* on *A* shows a peak, which implies the typical feature of noise-induced resonance (Fig. 5a). For *σ* = 1, the destructive role of *A* on the formation of spiral wave is restricted partly (Fig. 5a). Furthermore, when we set *σ* = 1, frequency maybe has no clear effect on the spiral wave in the currently used parameters (Fig. 5b). As a whole, the increased amplitude *A* let *R* increases monotonically (Fig. 5a,b). Therefore, the increased amplitude *A* tends to destroy spiral wave.

### Influences of frequency on formation and instability of spiral wave

**Figure 6:**
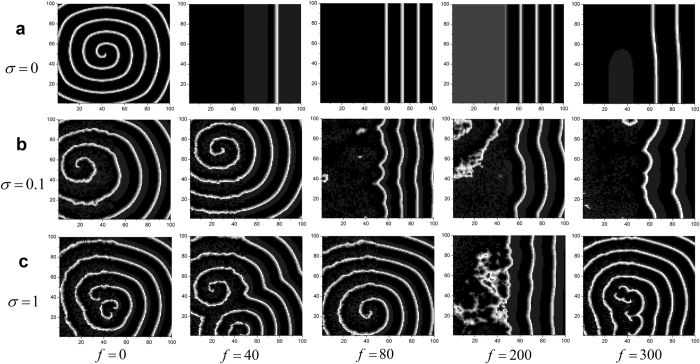
Developed patterns in the network at *t* = 500 time units, which are obtained by adjusting frequency *f* for a fixed amplitude *A* = 20 and the different intensities of (**a**) *σ* = 0; (**b**) *σ* = 0.1, and (**c**) *σ* = 1.

**Figure 7:**
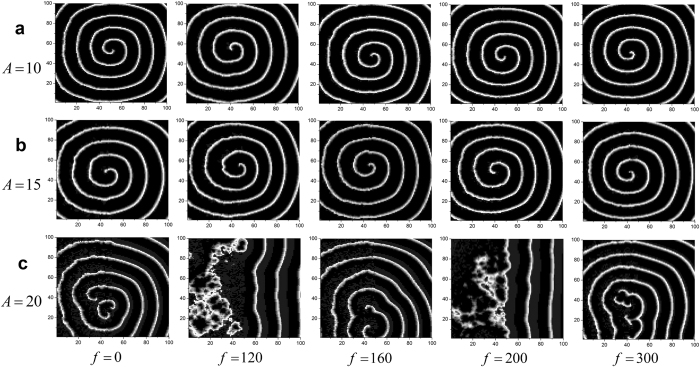
Developed patterns in the network at *t* = 500 time units, which are obtained by adjusting frequency *f* for a fixed intensity *σ* = 1 and the different amplitudes of (**a**) *A* = 10; (**b**) *A* = 15 and (**c**) *A* = 20.

**Figure 8:**
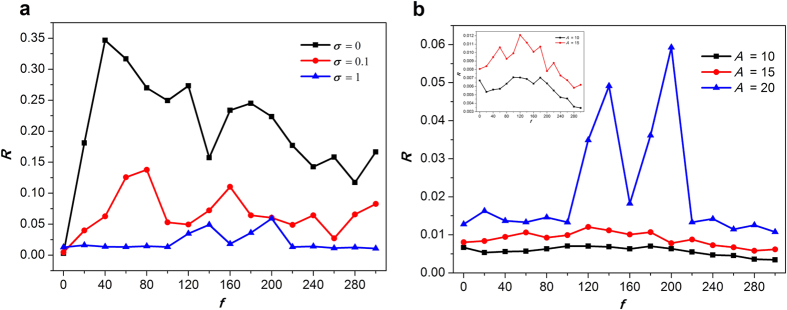
Synchronization factor *R* as a function of frequency *f* (**a**) for a fixed amplitude *A* = 20 and the different intensities; (**b**) for a fixed intensity *σ* = 1 and the different amplitudes. We set sufficiently large 500 time units in the calculation of *R*.

The bounded noise is also dependent on the frequency, it is important to evaluate the influences of frequency on formation and instability of spiral wave. In this case, we discuss how frequency influences wave formation and instability in the network, and the results are shown in Fig. 6 when we set *A* = 20. When *f* is in low value, one can observe the whole pattern of spiral wave. And the spiral wave gradually breaks by the increase of frequency, while the broken pattern can be organized again to form spiral wave if *σ* is large enough, e.g. 1 (Fig. 6). From the vertical perspective, one can also come to a conclusion that *σ* is also in favor of formation of spiral wave when frequency is increased (Fig. 6). In contrast, the increased amplitude destroys spiral wave (Fig. 7). For lower amplitude, such as 10 or 15, the increased frequency almost has no effect on the pattern of spiral wave (Fig. 7). Nevertheless, this conclusion may change for greater amplitude. For *A* = 20, we can observe a clear transition from formation to breakup, and from breakup to re-formation of spiral wave (Fig. 7). For each *σ* and *A, R*–*f* curve has a peak when *f* increases, which quantitatively characterize this transitions of patterns from ordered to disordered, and from disordered to ordered (Figs 6, 7 and 8). As depicted in Fig. 8a, larger *σ* corresponds to lower *R*, which indicates that *σ* has constructive role in formation of spiral wave, while larger *A* is linked to greater *R* (Fig. 8b), which manifests that increased amplitude tends to break spiral wave.

## Discussions and Conclusions

**Figure 9:**
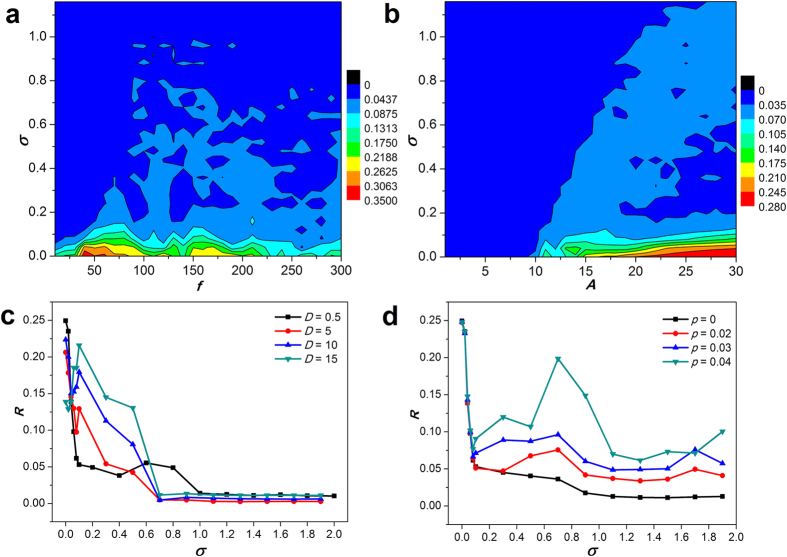
Contour plots and distributions of synchronization factor *R*. (**a**) Contour plot of *R* in the *σ* − *f* plane; (**b**) contour plot of *R* in the *σ* − *A* plane; (**c**) *R* as a function of *σ* for the different coupling strength *D* in 2D lattice network; (**d**) *R* as a function of *σ* for the different rewiring probability *p* in the SW network. The other fixed parameters used here are *A* = 20 for (**a**), *f* = 100 for (**b**), *A* = 20 and *f* = 100 for (**c**) and (**d**). We set sufficiently large 500 time units in the calculation of *R*.

It is also interesting and important whether the aforementioned results are valid for the wider parameter range of bounded noise. To further give a better global view, the contour plots of *R* in the *σ* − *f* and *σ* − *A* planes are depicted. When *f* is fixed, the increasing *σ* can significantly reduces *R*, which shows the constructive role of *σ* in the formation of spiral wave (Fig. 9a). In addition, *R* varies with *f* when *σ* is fixed, the plot of *R* versus *f* presents resonance-like phenomena for the appropriate *σ* (Fig. 9a). It is suggested that the frequency of bounded noise can play constructive or destructive role in pattern synchronization. The area with larger *R* value appears in the lower right corner of the contour plot of *R*, which indicates the disappearance of spiral wave by the increasing *A* (Fig. 9b). When *A* remains unchanged, augmenting *σ* lets spiral wave appear again. And the constructive role of *σ* in the formation of spiral wave is more prominent and clear for larger *A* value, such as 30. Furthermore, for larger *A* value, with the increase of *σ, R* firstly decreases rapidly, and then increases slowly, which presents the appearance of resonance-like phenomenons (Fig. 9b). It is worth mentioning that these results are in accord with results from specific parameters. Here *σ. f* and *A* of bounded noise cover very wider regions, which suggests that the above-mentioned results are robust against parameter perturbation.

In what follows, we discussed whether results hold unchanged under the different coupling strength and topology of neural networks. We firstly explored the influences of coupling strength *D*. When *D* is increased from 0.5 to 15, the curves of *R* − *σ* are close to each other for the larger *σ*, and the overall trends of curves almost remain unchanged (Fig. 9c). The nearest-neighbor connection in 2D square lattice seems simple and unrealistic. Human brain networks may share small-world (SW) topologies^44^,^45^. A SW network presents clear clustered structure and sparsely long-range random connectivity by rewiring connection between neurons with probability *p*, which can maximize the complexity or adaptivity of function which it can support while also minimizing costs^45^. Here SW networks are constructed to investigate the influences of coupling topology according to previous procedure^7^. Increasing *σ* also tends to induce the formation of spiral wave in SW networks (Fig. 9d). Moreover, increasing rewiring probability *p* leads to augment of *R*, thus destroy spiral wave for sufficiently large *p* by introducing the long-range random connection in the regular network (Fig. 9d). Therefore, our results are valid for more complex and realistic network structure.

As described above, bounded noise is able to model the random process which has either broad-band or narrow-band spectrum by appropriately tuning relevant parameters. Thus, for modeling the real physical system, bounded noise with bounded power is more practicable than white noise with the chance of taking large values. Although many interesting studies on bounded noise have been published, there are no results concerning the effect of bounded noise on the formation and instability of spiral wave in neuronal networks till now. To this purpose, we firstly present a regular 2D lattice neuronal networks, which is locally modeled by H-H neurons that located in the left hemi lattice is driven by bounded noise, and then investigate the effect of bounded noise by respectively adjusting the intensity *σ*, amplitude *A*, and frequency *f* of bounded noise. The numerical results confirmed that the increased intensity *σ* can facilitate the formation of spiral wave. Moreover, the increased amplitude tends to destroy spiral wave. Therefore, the parameter combination of appropriate amplitude and intensity may result in resonance-like phenomenon. In addition, when frequency is below a certain threshold, the increased frequency may let spiral wave instability, and yet it may result in formation of spiral wave for large enough intensity. Thus, the curve of *R* vs. *f* shows a peak quantitatively. In other words, resonance-like phenomenon also occurs by appropriately adjusting the parameters of frequency and intensity. Finally, the combination of frequency and amplitude also generate this transition of patterns from ordered to disordered, and further from disordered to ordered, which is illuminated by the two peaks of *R* curve. Taken together, the results of this study are not only beneficial to explore the effect of noise on the spiral wave in the nervous system, but also lay foundations for further research of other biological systems with bounded noise.

## Additional Information

**How to cite this article**: Yao, Y. *et al*. Impact of bounded noise on the formation and instability of spiral wave in a 2D Lattice of neurons. *Sci. Rep.* **7**, 43151; doi: 10.1038/srep43151 (2017).

**Publisher's note:** Springer Nature remains neutral with regard to jurisdictional claims in published maps and institutional affiliations.
